# Necrotizing fasciitis caused by *Haemophilus influenzae* type b in a patient with rectal cancer treated with combined bevacizumab and chemotherapy: a case report

**DOI:** 10.1186/1471-2334-14-198

**Published:** 2014-04-12

**Authors:** Tomotaka Ugai, Masataro Norizuki, Takahiro Mikawa, Goh Ohji, Makito Yaegashi

**Affiliations:** 1 Division of General Internal Medicine and Infectious Disease, Department of Medicine, Kameda Medical Center, Kamogawa-shi, Chiba 296-8601, Japan; 2 Current address; Division of Hematology, Department of Medicine, Saitama Medical Center, Jichi Medical University, Amanuma-cho, Omiya, Saitama-shi, Saitama 330-8503, Japan; 3 Current address; Department of Infectious Disease, Jichi Medical University, Shimotsuke, Tochigi 329-0498, Japan; 4 Division of Infectious Disease, Department of Microbiology and Infectious Diseases, Kobe University Graduate School of Medicine, 7-5-2 Kusunoki-cho, Chuo-ku, Kobe, Hyogo 650-0017, Japan

**Keywords:** Necrotizing fasciitis, *Haemophilus influenzae* type b, Bevacizumab

## Abstract

**Background:**

Recently, necrotizing fasciitis has been reported in patients treated with bevacizumab, usually secondary to wound healing complications, gastrointestinal perforations, or fistula formation. The risk of invasive *Haemophilus influenzae* type b infection is significantly increased in immunocompromised hosts. However, necrotizing fasciitis due to *Haemophilus influenzae* type b in a patient treated with combined bevacizumab and chemotherapy has not been previously reported.

**Case presentation:**

A 59-year-old woman was admitted to the intensive care unit after sudden onset of fever, chills, and right thigh pain. She received chemotherapy with fluorouracil, irinotecan, and bevacizumab for colon cancer 10 days prior to admission. The advancing erythematous margin and her worsening clinical condition prompted us to suspect necrotizing fasciitis and consult the orthopedics department for a fascia biopsy and debridement. Surgical exploration revealed a murky dishwater-colored pus exudate from the incision site and the lack of a shiny appearance of the fascia that also suggested necrotizing fasciitis. After 2 days, the final results of the blood and exudate cultures confirmed the presence of *Haemophilus influenzae* type b. A diagnosis of necrotizing fasciitis due to *Haemophilus influenzae* type b was made. The patient required recurrent surgical debridement and drainage, but she recovered from the septic shock.

**Conclusions:**

We report a case of necrotizing fasciitis due to *Haemophilus influenzae* type b in a patient without injury and with rectal cancer treated with combined bevacizumab and chemotherapy. Physicians should consider invasive *Haemophilus influenzae* type b disease in the presence of necrotizing fasciitis in patients treated with this combined treatment modality.

## Background

Bevacizumab is an antibody designed to inhibit vascular endothelial growth factor, which is involved in blood vessel formation, and used primarily in the cancer field for the treatment of colorectal cancer to prevent the formation of blood vessels that feed tumors. Recently, necrotizing fasciitis has been reported in patients treated with bevacizumab, including some fatal cases [1]. However, necrotizing fasciitis usually develops secondary to wound healing complications, gastrointestinal perforations, or fistula formation. Here, we report a case of necrotizing fasciitis due to *Haemophilus influenzae* type b (Hib) in a patient without injury and with rectal cancer treated with combined bevacizumab and chemotherapy.

### Case presentation

A 59-year-old woman was admitted to the intensive care unit after sudden onset of fever, chills, and right thigh pain. She had undergone low anterior resection for colon cancer 2 years earlier. After local recurrence, she underwent radiation therapy (30 Gy) for spinal metastasis followed by chemotherapy, which commenced 6 months prior to admission to the intensive care unit. She received chemotherapy with fluorouracil, irinotecan, and bevacizumab 10 days prior to admission. She did not have a history of previous injury or the presence of other risk factors for necrotizing fasciitis such as a decubitus ulcer, diabetes, or liver cirrhosis. Her medications included a 5-HT3 receptor antagonist, which is an antiemetic, and a proton pump inhibitor; however, her medications did not include non-steroidal anti-inflammatory drugs, immunosuppressive drugs, or corticosteroids.

She was hypotensive, tachycardic, and tachypneic in the emergency room. The results of the physical examination were normal except for erythema in her right thigh. She had no previous injuries. Hemoglobin level was 118 g/L with a white blood cell count of 2.1 × 10^9^ cells/L and a platelet count of 1.3 × 10^11^ cells/L. Blood urea nitrogen was 18.9 mmol/L, creatinine was 318.2 μmol/L, and C-reactive protein was 3.8 × 10^6^ μg/L. The results of other blood tests and urinalysis were normal. Computed tomography of the chest, abdomen, and pelvis showed normal results. Septic shock was suspected and managed accordingly with supportive measures, such as the use of a vasopressor and supplemental oxygen. Blood cultures were collected, and empirical treatment with meropenem (1 g every 12 hours) and vancomycin (1 g daily) were initiated, adjusted to renal dysfunction. The advancing erythematous margin and her worsening clinical condition prompted us to suspect necrotizing fasciitis and consult the orthopedics department for a fascia biopsy and debridement. Surgical exploration revealed a murky dishwater-colored pus exudate from the incision site and the lack of a shiny appearance of the fascia that also suggested necrotizing fasciitis. The biopsy of the frozen section showed acute necrotizing suppurative inflammation, and extensive debridement was performed. Gram staining of the exudates from the infected site showed the presence of gram-negative rods. On the basis of all of this information, we formally diagnosed the patient with necrotizing fasciitis. On the following day, gram-negative rods were also isolated from the blood cultures. After 2 days, the final results of the blood and exudate cultures confirmed the presence of β-lactamase-negative Hib that was susceptible to ampicillin, cefotaxime, ciprofloxacin, and imipenem. The histopathological findings of the debrided tissue were compatible with a diagnosis of necrotizing fasciitis due to Hib.

The patient required recurrent surgical debridement and drainage, but she recovered from the septic shock. The treatment was changed to ampicillin, and she received intravenous ampicillin for a total of 51 days because of residual intermuscular abscesses. She recovered completely and was discharged from the hospital 65 days after admission. Necrotizing fasciitis did not recur; however, she died of metastatic colorectal cancer 6 months after discharge.

## Discussion

Necrotizing fasciitis is a rare but life-threatening infection of the soft tissue that is characterized by rapidly spreading necrosis of the superficial fascia and subcutaneous tissue. Immunocompromised and diabetic patients are at a higher risk of developing necrotizing fasciitis [2]. Although bevacizumab suppresses the immune system [3], chemotherapy administered concurrently with bevacizumab is more likely to be responsible for immune suppression. This combined treatment also contributes to impaired wound healing. Furthermore, one of the pathophysiologic mechanisms of necrotizing fasciitis is subcutaneous artery thrombosis and tissue ischemia, to which bevacizumab can contribute [4]. Therefore, the combined treatment modality of bevacizumab and chemotherapy is likely to place patients at an increased risk of developing necrotizing fasciitis. Despite the fact that necrotizing fasciitis is a rare complication, affecting only 1 in 5000 bevacizumab users, physicians should bear in mind the risk of necrotizing fasciitis when prescribing bevacizumab.

In a comprehensive safety review conducted by the company Roche, 52 case reports of serious necrotizing fasciitis were identified that occurred between November 1997 and September 2012 in patients treated with bevacizumab for cancer [5]. The majority of the patients described in case reports have had gastrointestinal perforations, fistula formation, or wound healing complications preceding the development of necrotizing fasciitis [6-8]. However, in the present case, the patient had no previous injury or risk factor for necrotizing fasciitis such as a decubitus ulcer, diabetes, or liver cirrhosis; her recent chemotherapy was her primary risk for necrotizing fasciitis. These findings suggest a possible association between necrotizing fasciitis and the recent combined treatment of chemotherapy and bevacizumab in the absence of a previous injury.

It is of particular interest that Hib caused the necrotizing fasciitis in the present case. Prior to routine Hib vaccination, Hib was a well-known cause of invasive diseases, such as meningitis and pneumonia with bacteremia, in children younger than 2 years. However, the incidence of invasive *H. influenzae* disease, especially among persons aged over 65 years, and invasive non-typable *H. influenzae* disease has increased from 1996 to 2004 [9]. The risk of invasive Hib infection in adults is significantly increased by multiple myeloma and chronic renal failure [10]. Other factors conferring increased risk for *H. influenzae* infection include underlying immunocompromising conditions such as complement deficiency, hypogammaglobulinemia, sickle cell anemia, functional asplenia, malignancy, and human immunodeficiency virus infection [11,12]. Although the association between bevacizumab and invasive Hib infection remains unclear, we caution physicians to consider invasive Hib disease in patients treated with the combination of bevacizumab and chemotherapy.

A review of the current literature resulted in only 4 reported cases of necrotizing fasciitis caused by Hib. The first reported case occurred in a 13-month-old infant [13], while the second case occurred in an 81-year-old man with diabetes mellitus [14]. A 64-year-old woman who developed necrotizing fasciitis of her chest wall secondary to the epiglottitis represented the third case [15]. In the fourth case, a 44-year-old man developed necrotizing fasciitis of the right lower extremity after intramuscular injections of paracetamol in his right buttock [16]. Including our case, all of the patients with necrotizing fasciitis due to Hib made a full recovery. Hib merits additional consideration when deciding on appropriate, empiric antimicrobial therapy as an adjunct to surgical intervention for necrotizing fasciitis, especially in immunocompromised patients treated with combined bevacizumab and chemotherapy.

## Conclusions

We report a case of necrotizing fasciitis due to Hib in a patient without injury and with rectal cancer treated with combined bevacizumab and chemotherapy. Physicians should consider invasive Hib disease in the presence of necrotizing fasciitis in patients treated with this combined treatment modality.

## Consent

Written informed consent was obtained from the patient for publication of this case report. A copy of the written consent is available for review by the Editor-in-Chief of this journal.

## Abbreviations

Hib: *Haemophilus influenzae* type b.

## Competing interests

The authors declare that they have no competing interests.

All authors have submitted the ICMJE Form for Disclosure of Potential Conflicts of Interest. Conflicts that the editors consider relevant to the content of the manuscript have been disclosed.

## Authors’ contributions

All authors were involved in the clinical care of the patient and acquisition and interpretation of the data. TU drafted the manuscript. All authors read and approved the final manuscript.

## Pre-publication history

The pre-publication history for this paper can be accessed here:

http://www.biomedcentral.com/1471-2334/14/198/prepub
